# Message framing effects on attitude and intention toward social participation in old age

**DOI:** 10.1186/s12889-023-16555-1

**Published:** 2023-09-04

**Authors:** Hiroshi Murayama, Shusaku Sasaki, Yuta Takahashi, Mai Takase, Atsuko Taguchi

**Affiliations:** 1 Research Team for Social Participation and Healthy Aging, Tokyo Metropolitan Institute for Geriatrics and Gerontology, 35-2 Sakae-Cho, Itabashi, Tokyo 173-0015 Japan; 2 Center for Infectious Disease Education and Research (CiDER), Osaka University, 2-8 Yamadaoka, Suita, Osaka 565-0871 Japan; 3 Policy Bureau, City of Yokohama, 6-50-10 Hon-Cho, Naka-Ku, Yokohama, Kanagawa 231-0005 Japan; 4 Faculty of Nursing and Medical Care, Keio University, 4411 Endo, Fujisawa, Kanagawa 252-0883 Japan

**Keywords:** Message framing, Social participation, Attitude, Intention, Old age, Japan

## Abstract

**Background:**

Message framing is frequently used to advocate health perceptions and behaviors. The effects of message framing on various health behaviors have been examined; however, its effects on social participation, a key determinant of healthy aging, are unclear. This study investigated the effects of message framing on older adults’ attitudes and intentions toward social participation.

**Methods:**

A questionnaire survey conducted in 2020 targeted community-dwelling people aged ≥ 65 years in two rural areas in Japan. Participants were randomly allocated to four groups according to the types of framed messages to promote social participation activities: “private gain-framed message,” “private loss-framed message,” “public gain-framed message,” or “no message.” Outcomes included attitudes and intentions toward social participation (impression, interest, and readiness for social participation activities).

**Results:**

A total of 1,524 participants were analyzed (men: 46.3%; average age: 75.7 ± 7.9 years). Ordinal logistic regression analyses of individuals who engaged in any social participation activity showed no significant intergroup difference in the outcomes after adjusting for potential covariates. Among people who did not engage in any activity, the private loss-framed message was associated with a more favorable impression and higher interest and readiness than no message. The private gain-framed message was related to a higher interest in social participation.

**Conclusions:**

Private loss-framed messages are possibly most effective in reinforcing attitudes and intentions toward social participation, particularly among individuals without social participation experience. These findings highlight the possibility of using a message-framing approach to promote social participation in older adults.

**Supplementary Information:**

The online version contains supplementary material available at 10.1186/s12889-023-16555-1.

## Background

Public health professionals frequently use persuasive messages to modulate people’s perceptions and behaviors. Health messages can be of two types: “gain-framed” (highlighting the advantages of compliance with a particular behavior) and “loss-framed” (emphasizing the disadvantages of non-compliance with a particular behavior). This categorization is based on the Prospect Theory in behavioral economics, which assumes that people value gains and losses differently [1]. The theory avers that people’s choices and behaviors are influenced by whether they are framed in terms of gain or loss, even if their substance is essentially equivalent. People are more willing to accept risks when they evaluate options in terms of associated costs, but act to avoid risks when the same options are described in terms of the associated benefits [2]. Rothman and Salovey, who developed the Prospect Theory, suggested that “the tendency to perceive the function of detection behaviors as illness detecting facilitates the persuasiveness of loss-framed appeals, whereas the tendency to perceive prevention behaviors as health enhancing facilitates the effectiveness of gain-framed appeals” (p. 13) [3].

To date, many studies have examined the hypothesis that was proposed by Rothman and Salovey by investigating the effects of gain- and loss-framed messages on various health behaviors. A meta-analysis focused on preventive behaviors that aimed at avoiding illness and deterioration (e.g., smoking, physical activity, and diet) revealed that gain-framed messages significantly advocated preventive behaviors compared with loss-framed messages, although the difference was remarkably small [4]. In contrast, another meta-analysis investigating framing effects on detection behaviors that aimed to reflect the presence or absence of risk (e.g., cancer screening and anti-human immunodeficiency virus testing) found a significant but weak advantage of loss-framed messages over gain-framed messages [5]. These meta-analyses support the hypothesis of Rothman and Salovey [3], despite concluding the considerably weaker-than-expected persuasiveness of framed messages to advocate health behaviors. Nonetheless, as the effectiveness of message framing empirically depends on the context such as regions [6–8], further investigation of specific outcomes and populations is needed.

Social participation, which is generally understood as an individual’s involvement in activities that facilitate social or community interactions [9], is known to be important in the context of healthy behaviors, especially in later life. For example, several studies reported that social participation was related to lower risks of mortality [10–13] and frailty onset [14–16], indicating a key determinant of healthy aging. However, social participation was not just included in the above-mentioned meta-analyses with regard to message framing [4, 5] but also in other meta-analyses [17, 18]. To the best of our knowledge, the effects of message framing on social participation are not well understood; however, such an examination may help develop strategies to encourage social participation. In Japan, which has one of the most rapidly aging populations in the world, social participation is widely recognized as a strategy for promoting health and preventing frailty in old age [19]. Therefore, understanding the impact of message framing on social participation could have practical implications.

This study aimed to investigate the effect of message framing on attitudes and intentions toward social participation in older Japanese adults. Social participation constitutes prevention, rather than detection. Therefore, based on Rothman and Salovey’s theory [3], we hypothesized that a gain-framing message could effectively strengthen attitudes and intentions regarding social participation. Moreover, Bukov et al. proposed three types of social participation depending on the resources shared by individuals: collective, productive, and political [20]. In this study, we focused on collective social participation—the common action of group members through shared time [20]—in formal and informal societal groups in the local community for activities such as neighborhood associations, senior clubs, and hobby and sports groups. Furthermore, it has been reported that older people living in rural areas are less active in social participation compared with those in urban areas [21]; hence, there may be potential to increase the number of individuals engaging in social participation activities in rural areas. Therefore, this study was conducted in rural communities.

## Methods

### Study design and participants

Data were obtained, between September and October 2020, from cross-sectional surveys of community-dwelling people aged ≥ 65 years without severe dependence and living in two rural areas of Japan (Gejo area in Tokamachi City, Niigata Prefecture, and Yoshijima area in Kawanishi Town, Yamagata Prefecture). On March 31, 2020, the population size (proportion) of people aged ≥ 65 years was 51,568 (39.1%) in Tokamachi and 14,901 (37.4%) in Kawanishi.

Gejo in Tokamachi comprised 1,229 older adults as of September 1, 2020. After excluding 127 individuals certified with care levels 3–5 in the long-term care insurance system and 40 who had died by the date of questionnaire distribution, the questionnaire was sent to 1,062 individuals by mail and 868 returned the questionnaires (response rate: 81.7%). As we excluded 19 respondents who were not living in their own homes (e.g., currently undergoing long-term hospitalization or residing in care facilities) or provided invalid responses (e.g., a blank questionnaire) and 14 who did not respond to the question on engagement in social participation activities, 835 participants were consequently included in the analysis.

Yoshijima in Kawanishi included 942 older adults as of July 31, 2020. As two older adults died before the survey commenced, we sent the questionnaires to 940 individuals by mail and received 803 completed questionnaires (response rate: 85.4%). After excluding 51 respondents who were not living in their own homes, were certified with care levels 3–5 in the long-term care insurance system, or provided invalid responses, and 63 who did not respond to the question on engagement in social participation activities, a total of 689 participants were included in the analysis.

The study protocol was approved by the ethics committee of the Tokyo Metropolitan Institute for Geriatrics and Gerontology (approved on August 20, 2020) and Tohoku University Graduate School of Medicine (approved on April 12, 2020). All participants provided informed consent prior to their inclusion in the study.

### Framed messages and allocation

In this study, three types of framed messages to promote social participation were used: i) private gain-framed messages, ii) private loss-framed messages, and iii) public gain-framed messages (Table 1). The private gain-framed message referred to the advantage of engagement in collective social participation activities for individuals’ self-fulfillment and medical/long-term care costs. In contrast, the private loss-framed message explained the disadvantage of non-engagement in collective social participation activities with regard to individuals’ self-fulfillment and medical/long-term care costs. These messages were prepared based on evidence from previous Japanese studies [12, 22, 23]. The public gain-framed message described the advantage of engagement in collective social participation activities for community connectedness and the security of residents in the community, based on evidence from previous Japanese studies [24–26].

**Table 1:** Three types of framed messages to promote social participation that were used in this study

Type	Message
Private gain-framed	“Social participation is known to have a positive impact on your own health and dementia prevention. If you engage in social participation activities, you can continue to do what you want for longer and reduce your medical and long-term care costs.”
Private loss-framed	“Social participation is known to have a positive impact on your own health and dementia prevention. If you do not engage in social participation activities, you may have to give up some of what you want and incur high medical and long-term care costs.”
Public gain-framed	“Social participation is known to have a positive impact on the community where you live. If you engage in social participation activities, community connectedness can be enhanced and the residents can continue to live in the community with security.”

Before conducting the survey, we randomly allocated the samples into four groups in the two study areas. For three groups, we presented each framed message on the questionnaire immediately before the questions on social participation (explained below). The participants answered the social participation questions after reading the framed message. The remaining group received no messages (i.e., control group). The participants in this group answered the social participation questions without reading any messages.

### Measures

#### Social participation

First, we asked whether the respondents engaged in any community-based social participation activities. The respondents answered “yes” or “no.” For people who participated in any activity, we further asked about the type of activity such as “neighborhood associations,” “senior clubs,” “volunteer groups,” “hobby groups, sports groups, or learning groups.”

Second, we asked about the respondent’s attitudes and intentions toward social participation as the outcome variables because previous studies regarding the effects of message framing on health-related outcomes focused on three domains (attitudes, intentions, and behaviors) [18] based on the behavioral theory (e.g., the Theory of Planned Behavior [27]). As this study was cross-sectional, we were unable to capture the effect of reading messages on behavioral change; therefore, only two domains were measured: attitudes and intentions. Attitudes toward social participation were assessed based on impressions of the social participation activity, as measured by the item: “What is your impression of social participation activities?” with answers scored on a five-point Likert scale (“1 = unfavorable,” “2 = somewhat unfavorable,” “3 = neither,” “4 = somewhat favorable,” or “5 = favorable”).

The intention for social participation includes an interest in and readiness for social participation activities. Interest in social participation activities was assessed using the item: “How interested are you in social participation activity now?” with answers scored using a six-point Likert scale (“1 = I am not interested in it at all,” “2 = I am not interested in it,” “3 = I am not much interested in it,” “4 = I am somewhat interested in it,” “5 = I am interested in it,” or “6 = I am very interested in”). Readiness for social participation activities was assessed after preparing items for those with and without participation in any social participation activity as “How would you like to change the frequency and variation of social participation activities you are currently engaged in in the future?” (scored using a three-point Likert scale: “1 = I would like to decrease the frequency and variation,” “2 = I would like to keep the current situation unchanged,” or “3 = I would like to increase the frequency and variation”) and “How much would you like to initiate social participation activities?” (scored using a three-point Likert scale: “1 = I will not begin activities,” “2 = I would eventually like to begin activities,” or “3 = I would like to begin activities in the near future”), respectively.

#### Covariates

Information on sex, age, marital status (“married” or “unmarried”), education (“high school graduation and higher” or “less”), subjective financial stability, working status (“currently working” or “not working”), neighborly tie, and self-rated health was obtained via the questionnaire as these variables have been reported to affect an individual’s perception of social participation [28, 29]. Subjective financial stability was assessed using a five-point Likert scale (“affluent,” “somewhat affluent,” “normal,” “somewhat poor,” or “poor”). Neighborly tie was assessed using a single item: “How is your relationship with your neighbors?” with answers scored on a four-point Likert scale (“I often talk with neighbors about my problems,” “I only make small talk with my neighbors,” “I only greet my neighbors” or “I am not friendly with my neighbors”). Self-rated health was measured on a four-point Likert scale (“good,” “somewhat good,” “somewhat poor,” or “poor”). Besides these variables, the study area (i.e., “Gejo in Tokamachi” or “Yoshijima in Kawanishi”) was included as a covariate.

### Statistical analysis

First, using the chi-square test, one-way analysis of variance, and the Kruskal–Wallis test, we compared the participants’ characteristics among the four groups that were stratified according to message allocation. Second, ordinal logistic regression analyses were conducted to examine intergroup differences in attitudes and intentions. We performed the analyses separately according to engagement or non-engagement in social participation activities by using a two-step modeling strategy: Model 1, without adjustment, and Model 2, with covariates fully controlled. For people with engagement in social participation activities, the type of activity was also controlled in Model 2. The results are presented as odds ratios (ORs) with 95% confidence intervals (CIs). All analyses were performed using IBM SPSS Statistics 29 (IBM Corp., Armonk, NY, USA).

## Results

Table 2 presents the participants’ descriptive characteristics. Among the 1,524 participants who were included in the analysis (835 from Gejo and 689 from Yoshijima), 46.3% were men, the average age was 75.7 years (standard deviation: 7.9 years), and 71.8% were married. In terms of socioeconomic status, 54.6% graduated from high school and over, approximately 10% felt affluent, and 33.2% had a job. Moreover, approximately 73% had strong neighborly ties (i.e., the responses of “I often talk with neighbors about my problems” and “I only make small talk with my neighbors”), and 67% recognized that they had good health conditions. Regarding social participation activities, 53.6% (*n* = 817) and 46.4% (*n* = 707) did and did not engage in any activity, respectively. Among those who engaged in social participation activities, 37.9%, 29.7%, 17.8%, and 45.5% participated in neighborhood associations, senior clubs, volunteer groups, and hobby, sports, or learning groups, respectively (data not shown in the table). Intergroup differences in marital status were statistically significant (*p* = 0.013).

**Table 2:** Descriptive characteristics by the message allocation

	Total (*n* = 1,524)	Private gain-framed message (*n* = 394)	Private loss-framed message (*n* = 379)	Public gain-framed message (*n* = 372)	No message (*n* = 379)	*P*
Study area						
Gejo in Tokamachi	54.8	54.3	55.9	53.8	55.1	0.937^a^
Yoshijima in Kawanishi	45.2	45.7	44.1	46.2	44.9	
Sex						
Men	46.3	47.0	43.0	45.4	49.6	0.321^a^
Age (years)						
	75.7 ± 7.9	75.7 ± 8.1	75.8 ± 7.9	76.1 ± 7.9	75.3 ± 7.7	0.630^b^
65–69	26.4	26.2	25.3	26.1	28.0	0.724^c^
70–79	41.5	42.0	42.0	40.1	42.2	
80–89	26.4	25.2	27.4	28.5	24.5	
≥ 90	5.6	6.6	5.3	5.4	5.3	
Marital status						
Married	71.8	77.8	71.4	67.6	70.1	0.013^a^
Education						
≥ High school graduation	54.6	56.7	52.4	55.4	53.8	0.659^a^
Subjective financial stability						
Affluent	2.5	2.3	2.4	3.0	2.1	0.631^c^
Somewhat affluent	7.3	7.2	7.4	7.7	6.9	
Normal	65.3	63.8	66.3	65.7	65.4	
Somewhat poor	19.0	19.0	17.5	19.2	20.2	
Poor	6.0	7.7	6.4	4.4	5.3	
Working status						
Currently working	33.2	38.2	31.3	29.4	33.6	0.064^a^
Neighborly tie						
I often talk with neighbors about my problems	23.2	24.2	22.0	23.8	23.0	0.871^c^
I only make small talk with my neighbors	49.5	46.8	51.2	47.6	52.4	
I only greet my neighbors	23.6	24.9	23.3	24.3	21.7	
I am not friendly with my neighbors	3.7	4.1	3.5	4.3	2.9	
Self-rated health						
Good	3.3	3.6	1.6	3.3	4.9	0.362^c^
Somewhat good	63.7	62.6	66.4	61.5	64.4	
Somewhat poor	23.7	26.2	23.3	22.7	22.6	
Poor	9.2	7.7	8.7	12.6	8.1	
Social participation						
Engaging in any social participation activity	53.6	58.1	52.0	51.3	52.8	0.212^a^

Values represent the proportion or mean ± standard deviation

^a^chi-square test

^b^t-test

^c^Kruskal–Wallis test

Table 3 presents the outcome distribution according to engagement in social participation activity. People who were already engaged in social participation activities tended to have a more favorable impression and higher interest in social participation than those who were not. In terms of readiness for social participation activities, approximately 78% of people who engaged in social participation activities and 73% of people who did not respond that they would like to maintain their status quo.

**Table 3:** Descriptive characteristics of the outcomes by engagement in social participation activities

		Engaging in any social participation activity (*n* = 817)	Not engaging in any social participation activity (*n* = 707)
Impression of social participation activity	Unfavorable (= 1)	0.0	0.0
	Somewhat unfavorable (= 2)	1.6	1.2
	Neither (= 3)	25.9	58.2
	Somewhat favorable (= 4)	54.2	32.2
	Favorable (= 5)	18.2	8.3
		3.9 ± 0.7	3.5 ± 0.7
Interest in social participation activity	I am not interested in it at all (= 1)	0.3	7.9
	I am not interested in it (= 2)	2.0	15.0
	I am not much interested in it (= 3)	15.6	42.6
	I am somewhat interested in it (= 4)	45.4	23.3
	I am interested in it (= 5)	30.9	10.5
	I am very interested in it (= 6)	5.9	0.8
		4.2 ± 0.9	3.2 ± 1.1
Readiness for social participation activity	I would like to decrease the frequency and variation (= 1)	14.3	-
	I would like to keep the current situation unchanged (= 2)	78.3	-
	I would like to increase the frequency and variation (= 3)	7.4	-
		1.9 ± 0.5	-
	I will not begin activities (= 1)	-	72.9
	I would eventually like to begin activities (= 2)	-	26.3
	I would like to begin activities in near future (= 3)	-	0.8
		-	1.3 ± 0.5

Values represent the proportion or mean ± standard deviation

Tables 4 and 5 present the results of the ordinal logistic regression analyses conducted to examine the association between framed messages and outcomes. There was no significant intergroup difference in the outcome variables among people with engagement in social participation activity (Table 4). In contrast, some differences were detected among participants who did not engage in any social participation activity (Table 5). The favorable impression of social participation was greater in the private loss-framed message group than in the no-message group in Model 1. This association remained unchanged after adjusting for covariates in Model 2 (OR [95% CI] = 1.58 [1.03–2.43] and 1.55 [1.01–2.44] in Models 1 and 2, respectively). Thus, people who read the private loss-framed message had a more favorable impression of social participation activities. Interest in social participation activities was greater among the private gain-framed and private loss-framed message groups in both Models 1 and 2 (e.g., OR [95% CI] = 1.69 [1.10–2.58] for the private gain-framed message and 1.96 [1.30–2.95] for the private loss-framed message, in Model 2). The private loss-framed message group showed statistically significant readiness for social participation activities in Model 2 (OR [95% CI] = 1.84 [1.03–3.28]).

**Table 4:** Association of framed messages with attitude and intention toward social participation among older adults who were engaged in social participation activities

	More favorable impression of social participation activity	Higher interest in social participation activity	Higher readiness for increasing frequency and variety of social participation activity
	OR (95% CI)	OR (95% CI)	OR (95% CI)
Model 1 (crude)			
Private gain-framed message	1.11 (0.77–1.61)	0.99 (0.70–1.42)	1.62 (1.00–2.62)
Private loss-framed message	1.44 (0.98–2.12)	1.28 (0.89–1.86)	1.51 (0.92–2.48)
Public gain-framed message	1.12 (0.76–1.65)	1.16 (0.80–1.69)	0.82 (0.51–1.33)
No message	Ref	Ref	Ref
Model 2 (covariates-adjusted)			
Private gain-framed message	1.18 (0.79–1.77)	1.01 (0.69–1.48)	1.56 (0.94–2.58)
Private loss-framed message	1.38 (0.92–2.09)	1.42 (0.96–2.10)	1.34 (0.80–2.24)
Public gain-framed message	1.08 (0.71–1.63)	1.11 (0.75–1.65)	0.71 (0.43–1.18)
No message	Ref	Ref	Ref

Sex, age, marital status, education, financial stability, working status, neighborly ties, self-rated health, study area, and the type of activity were adjusted in Model 2

*CI* Confidence interval, *OR* Odds ratio

**Table 5:** Association of framed messages with attitude and intention toward social participation among older adults who had not engaged in social participation activities

	More favorable impression of social participation activity	Higher interest in social participation activity	Higher readiness for beginning social participation activity
	OR (95% CI)	OR (95% CI)	OR (95% CI)
Model 1 (crude)			
Private gain-framed message	1.29 (0.83–2.02)	1.57 (1.05–2.35)	0.97 (0.58–1.62)
Private loss-framed message	1.58 (1.03–2.43)	1.86 (1.26–2.76)	1.33 (0.82–2.14)
Public gain-framed message	1.31 (0.85–2.04)	1.27 (0.85–1.88)	0.83 (0.50–1.37)
No message	Ref	Ref	Ref
Model 2 (covariates-adjusted)			
Private gain-framed message	1.29 (0.80–2.06)	1.69 (1.10–2.58)	1.34 (0.73–2.46)
Private loss-framed message	1.55 (1.01–2.44)	1.96 (1.30–2.95)	1.84 (1.03–3.28)
Public gain-framed message	1.39 (0.87–2.21)	1.49 (0.96–2.26)	1.18 (0.64–2.19)
No message	Ref	Ref	Ref

Sex, age, marital status, education, financial stability, working status, neighborly ties, self-rated health, and study area were adjusted in Model 2

*CI* Confidence interval, *OR* Odds ratio

For a sensitivity analysis, we performed ordinal logistic regression analyses, while setting the private loss-framed message as the reference (Supplementary Tables 1 and 2). No significant difference was observed in the outcome variables between the private gain-framed and private loss-framed messages.

## Discussion

This study examined the effects of message framing on attitudes and intentions regarding social participation among older Japanese people living in rural areas. The private gain- and loss-framed messages tended to be more effective in increasing attitude and intention toward social participation than no messages, particularly among people without social participation activities. Although a statistically significant difference between the private gain- and loss-framed messages was not detected, we found that the private loss-framed message was possibly most effective at promoting social participation in older adults. Several studies have examined the effect of message framing on various health behaviors, including smoking, physical activity, dietary habits, and cancer screening, whereas no study has focused on social participation.

We hypothesized that a gain-framed message would be the most effective in promoting attitudes and intentions toward social participation based on previous research [3–5]. However, contrary to our hypothesis, those who read the private loss-framed message had the highest attitude and intention toward social participation, particularly those who had not engaged in social participation activities. To date, several factors have been evident in cases where gain- or loss-framing is effective [30, 31]. One is “a certainty of outcome,” which pertains to the perception of whether the potential health consequence related to a specific behavior is certain or uncertain. Loss-framed messaging could be more effective if health consequences are uncertain. Moreover, certainty (or uncertainty) depends on the individuals’ recognition of loss rather than objective evidence [32]. For those who were not engaged in any social participation activities, it might have been difficult to realize the health influence of social participation; therefore, they seemed to recognize it as uncertain. Thus, the private loss-framed message might strongly reinforce attitudes and intentions toward social participation.

Another factor is “the level of involvement,” which refers to a person’s involvement, understanding, or interest in a particular issue (i.e., social participation activities in this case). Gain-framed messaging is effective for those with low involvement whereas loss-framed messaging is effective for those with high involvement in the issue [3]. Our results showed that, besides the private loss-framed message, the private gain-framed message was associated with higher interest than the control group among participants without engagement in social participation activities. People who are highly involved in an issue are likely to have specific knowledge that enables them to respond to fear-based messages that activate their loss-aversion response [3]. In contrast, those who are uninvolved in the issue lack sufficient detailed knowledge to respond to fear-based messages and tend to pay more attention to gain-framed positive messages [33]. Therefore, the gain-framed message was linked to greater interest among people without social participation experience.

A meta-analysis conducted by Gallagher and Updegraff separately examined the impact of message framing on attitudes and intentions. Their findings concluded that there was no difference in the effect of gain- and loss-framed messages on attitudes and intentions for preventive behaviors, including smoking, physical activity, and diet [18]. They reported that the effect sizes, which summarized the comparison of the effect between gain- and loss-framed messages, were weak (less than 0.1) and statistically nonsignificant for both attitudes and intentions toward preventive behaviors, at 0.039 and 0.028, respectively [18]. This study focused on attitude and intention outcomes in terms of social participation and found some effects of message framing (particularly the private loss-framed message) on the outcomes. The effect of message framing on social participation has not been included in previous meta-analyses [4, 5, 17, 18] or investigated yet. The mechanism associated with message framing that influences social participation might differ from other health behaviors that have been discussed previously. Therefore, further research on this topic is required.

In contrast to the results observed among those who were not engaged in social participation activities, neither gain- nor loss-framed messages showed a significant association with attitude or intention outcomes among individuals with social participation experiences. Since many participants who participated in activities had already developed high attitudes and intentions toward social participation, the effect of the message might have been underestimated in the current sample. Furthermore, as strong attitudes and intentions are generally considered psychological antecedents to behavioral change, their attitudes and intentions toward social participation may have already been reinforced. Therefore, the framing message might prove ineffective among those who have already engaged in social participation activities.

In this study, we prepared two types of gain-framed messages: private and public. However, the public gain-framed message was not associated with any outcome variable. This message was intended to appeal to people’s altruism because several studies have indicated that altruistic motivation may promote healthy behaviors [34–36]. However, with regard to social participation, altruism could not be a motivation. Moreover, the study was performed in rural areas with close-knit community ties: approximately 73% of the sample had strong neighborly ties, whereas a national survey indicated that approximately 65% of the general Japanese population had strong neighborly ties [37]. In such communities, there may be a discrepancy in community orientation between those who already participate in social activities and those who do not. This may have been the reason the message emphasizing public benefit (i.e., the advantage of activity engagement for community connectedness and the security of the residents in the whole community) was less effective than the one appealing to an individual’s benefit or loss.

This study had several limitations. First, this was an observational study that used data from questionnaire surveys; therefore, it is necessary to verify the findings in interventional studies to ascertain whether framed messages on social participation can prompt behavioral change (i.e., whether participants actually begin engaging in social participation activities or increase their frequency and types of activities). A meta-analysis highlighted a significant gap between attitude, intention, and behavior [38]. Therefore, while we focused on outcome variables related to attitudes and intentions toward social participation, it is possible that our findings may have overestimated the potential for behavioral change. Second, we employed three single items as the outcome measures. Although we based these items on domains used in previous studies [18], they were original to this research; hence, their reliability and validity have not been confirmed. In future studies, it is essential to use items with higher reliability and validity. Third, the surveys were conducted between September and October 2020, which was during the pandemic of the coronavirus disease 2019. The pandemic possibly influenced people’s perceptions and behaviors regarding social participation. Thus, the results of this study may have been affected by the pandemic and it cannot be ruled out that they differ from the results that may have been obtained under normal circumstances. Fourth, because we performed the survey in two rural areas of Japan, the generalizability of the findings is limited. Therefore, a cautious interpretation of these findings is recommended as social participation circumstances differ between urban and rural areas.

This study makes a significant contribution by uncovering the potential of framing messages to enhance social participation. While the importance of social participation for health promotion and frailty prevention in older people is widely recognized, an effective approach to encourage it has been lacking. As the cost of creating or modifying the messaging is low (sometimes zero), message framing may be cost-beneficial for promoting changes in people’s perceptions and behaviors. Furthermore, it is an easily implementable method. These constitute a significant advantage for the message-framing approach. Moreover, although the message-framing approach is not a panacea, our findings provide insight into developing effective approaches to facilitate older adults’ social participation. Our findings may instigate further interventions exploring the impact of message framing on social activity participation leading to valuable cumulative insights. Additionally, since the persuasive effect of message framing was particularly pronounced among those who were not engaged in any social participation activity, the intervention using framing messages may be highly effective for this specific group. Our results may be instrumental in identifying the target population for message-framing interventions.

## Conclusions

This study revealed that private loss-framed messages can effectively reinforce attitudes and intentions toward social participation in older adults. This was observed in people who did not engage in any social participation activity. Given the paucity of research on the effects of message framing on social participation, further research, especially on its behavioral effects, is needed. However, our findings highlight the possibility that a message-framing approach may promote social participation in older people.

### Supplementary Information

**Additional file 1: Supplementary Table 1.** Association of framed messages with attitude and intention regarding social participation among older adults who were engaged in social participation activities (setting the private loss-framed message as the reference). **Supplementary Table 2.** Association of framed messages with attitude and intention regarding social participation among older adults who were not engaged in social participation activities (setting the private loss-framed message as the reference).

## Data Availability

The datasets used and analyzed during the current study are available from the corresponding author on reasonable request.

## Abbreviations

CI: Confidence interval
OR: Odds ratio
