# Supplementary material for: Message framing effects on attitude and intention toward social participation in old age
Source: BMC Public Health. 2023 Sep 4;23:1713. doi: 10.1186/s12889-023-16555-1 (PMC10476306; doi:10.1186/s12889-023-16555-1)
Supplement: Supplementary file 1 — Additional file 1: Supplementary Table 1. Association of framed messages with attitude and intention regarding social participation among older adults who were engaged in social participation activities (setting the private loss-framed message as the reference). Supplementary Table 2. Association of framed messages with attitude and intention regarding social participation among older adults who were not engaged in social participation activities (setting the private loss-framed message as the reference). [file 12889_2023_16555_MOESM1_ESM.docx]

| **Supplementary Table 1.** Association of framed messages with attitude and intention regarding social participation among older adults who were engaged in social participation activities (setting the private loss-framed message as the reference) | | | | | |
| --- | --- | --- | --- | --- | --- |
|  | More favorable impression of social participation activity |  | Higher interest in social participation activity |  | Higher readiness for increasing frequency and variety of social participation activity |
|  | OR (95% CI) |  | OR (95% CI) |  | OR (95% CI) |
| Model 1 (crude) |  |  |  |  |  |
| Private gain-framed message | 0.77 (0.53–1.12) |  | 0.77 (0.54–1.11) |  | 1.07 (0.66–1.74) |
| Public gain-framed message | 0.78 (0.53–1.15) |  | 0.90 (0.62–1.32) |  | 0.54 (0.33–0.89) |
| No message | 0.69 (0.47–1.02) |  | 0.78 (0.54–1.13) |  | 0.66 (0.40–1.08) |
| Private loss-framed message | Ref. |  | Ref. |  | Ref. |
| Model 2 (covariates-adjusted) |  |  |  |  |  |
| Private gain-framed message | 0.86 (0.57–1.28) |  | 0.71 (0.49–1.04) |  | 1.16 (0.70–1.92) |
| Public gain-framed message | 0.78 (0.51–1.18) |  | 0.78 (0.53–1.16) |  | 0.53 (0.32–0.89) |
| No message | 0.72 (0.48–1.09) |  | 0.70 (0.48–1.04) |  | 0.75 (0.45–1.25) |
| Private loss-framed message | Ref. |  | Ref. |  | Ref. |
| CI: confidence interval. OR: odds ratio. | | | | | |
| Sex, age, marital status, education, financial stability, working status, self-rated health, study area, and the type of activity were adjusted in Model 2. | | | | | |

| **Supplementary Table 2.** Association of framed messages with attitude and intention regarding social participation among older adults who were not engaged in social participation activities (setting the private loss-framed message as the reference) | | | | | |
| --- | --- | --- | --- | --- | --- |
|  | More favorable impression of social participation activity |  | Higher interest in social participation activity |  | Higher readiness for beginning social participation activity |
|  | OR (95% CI) |  | OR (95% CI) |  | OR (95% CI) |
| Model 1 (crude) |  |  |  |  |  |
| Private gain-framed message | 0.82 (0.54–1.25) |  | 0.84 (0.57–1.25) |  | 0.73 (0.45–1.19) |
| Public gain-framed message | 0.83 (0.55–1.26) |  | 0.68 (0.46–1.00) |  | 0.62 (0.38–1.01) |
| No message | 0.63 (0.41–0.97) |  | 0.54 (0.36–0.79) |  | 0.75 (0.47–1.22) |
| Private loss-framed message | Ref. |  | Ref. |  | Ref. |
| Model 2 (covariates-adjusted) |  |  |  |  |  |
| Private gain-framed message | 0.77 (0.51–1.17) |  | 0.85 (0.56–1.28) |  | 0.77 (0.51–1.17) |
| Public gain-framed message | 0.72 (0.48–1.08) |  | 0.75 (0.50–1.12) |  | 0.72 (0.48–1.08) |
| No message | 0.50 (0.33–0.75) |  | 0.50 (0.33–0.76) |  | 0.50 (0.33–0.75) |
| Private loss-framed message | Ref. |  | Ref. |  | Ref. |
| CI: confidence interval. OR: odds ratio. | | | | | |
| Sex, age, marital status, education, financial stability, working status, self-rated health, and study area were adjusted in Model 2. | | | | | |
